# Trajectories of physical and mental functioning over 25 years before onset of frailty: results from the Whitehall II cohort study

**DOI:** 10.1002/jcsm.13129

**Published:** 2022-11-17

**Authors:** Benjamin Landré, Céline Ben Hassen, Mika Kivimaki, Mikaela Bloomberg, Aline Dugravot, Alexis Schniztler, Séverine Sabia, Archana Singh‐Manoux

**Affiliations:** ^1^ Université Paris Cité, Inserm U1153, CRESS, Epidemiology of Ageing and Neurodegenerative Diseases Paris France; ^2^ Department of Epidemiology and Public Health University College London London UK

**Keywords:** Frailty, Life course, Functioning, Epidemiology

## Abstract

**Background:**

Research on frailty, a major contributor to heterogeneity in health, is undertaken on older adults although the processes leading to frailty are likely to begin earlier in the life course. Using repeat data spanning 25 years, we examined changes in physical and mental functioning before the onset of frailty, defined using Fried's frailty phenotype (FFP).

**Methods:**

Functioning was measured using the Short‐Form 36 General Health Survey (SF‐36) on nine occasions from 1991 (age range 40–63 years) to 2015 (age range 63–85 years). The poorest of four FFP scores from 2002, 2007, 2012 and 2015 was used to classify participants as frail, pre‐frail, or robust. We used linear mixed models with a backward timescale such that time 0 was the person‐specific date of frailty classification for frail and pre‐frail participants and the end of follow‐up for robust participants. Analyses adjusted for socio‐demographic factors, health behaviours, body mass index and multi‐morbidity status were used to compare SF‐36 physical (PCS) and mental (MCS) component summary scores over 25 years before time 0 as a function of FFP classification, with estimates extracted at time 0, −5, −10, −15, −20 and −25 years. We also used illness–death models to examine the prospective association between SF‐36 component summary scores at age 50 and incident FFP‐defined frailty.

**Results:**

Among 7044 participants of the Whitehall II cohort study included in the analysis [29% female, mean age 49.7 (SD = 6.0) at baseline in 1991], 2055 (29%) participants remained robust, and 4476 (64%) became pre‐frail and 513 (7%) frail during follow‐up. Frail compared with robust participants had lower SF‐36 scores at *t* = −25 before onset of frailty with a difference of 3.4 [95% confidence interval (CI) 1.6, 5.1] in PCS and 1.8 (−0.2, 3.8) in MCS. At *t* = 0, the differences increased to 11.5 (10.5, 12.5) and 9.1 (8.0, 10.2), respectively. The differences in SF‐36 between the robust and pre‐frail groups, although smaller [at *t* = 0, 1.7 (1.2, 2.2) in PCS and 4.0 (3.4, 4.5) in MCS], were already observed 20 and 25 years, respectively, before the onset of pre‐frailty. Prospective analyses showed that at age 50, scores in the bottom quartiles of PCS [hazard ratio (HR) compared with the top quartile = 2.39, 95% CI 1.85, 3.07] and MCS [HR = 1.49 (1.15, 1.93)] were associated with a higher risk of FFP‐defined frailty at older ages.

**Conclusions:**

Differences in trajectories of physical and mental functioning in individuals who developed physical frailty at older ages were observable 25 years before onset of FFP‐defined frailty. These findings highlight the need for a life course approach in efforts to prevent frailty.

## Introduction

The concept of frailty, defined as a state of increased vulnerability to stressors, emerged in the gerontology literature to explain clinical heterogeneity in health of older adults.^1^ Current clinical practice guidelines recommend screening for frailty in all adults 65 years and older in the general population,^2^ with the World Health Organization advocating active case finding and reorientation of health services in individuals with frailty.^3^

Tools to measure frailty have been developed and used mostly on older adults, often older than 75 years. These include the Fried's frailty phenotype (FFP),^4^ which has also been used in intervention studies on frailty.^5^, ^6^, ^7^ Although the prevalence of frailty increases steadily with age, the processes underlying frailty are likely to begin well before old age. There is emerging evidence of frailty in middle‐aged adults,^8^, ^9^, ^10^ and the importance of a life course approach to frailty is increasingly recognized.^11^ Despite considerable research on frailty in recent years, little is known about the changes in physical and mental function leading to this syndrome.^11^, ^12^ Better understanding of these changes is likely to provide insight into optimal timing of screening and targeted therapeutic interventions and early prevention.

The objective of the present study was to examine whether deficits in physical and mental functioning are present before the onset of frailty, defined using FFP. Using data from the Whitehall II cohort study, we compared 25‐year trajectories of physical and mental functioning before the onset of FFP frailty. In complementary analyses, we also used prospective analyses to examine whether poor functioning at age 50 was associated with risk of FFP frailty at older ages.

## Methods

### Study population and design

The Whitehall II study is an ongoing prospective cohort study of 10 308 British civil servants, 6895 men and 3413 women, aged 35–55 in 1985–1988.^13^ Since baseline, follow‐up clinical examinations have taken place approximately every 4–5 years using home‐based assessment for those who choose this option and clinic‐based assessments (London and major cities in the UK) for others; each wave has taken approximately 2 years to complete. In addition to clinical examinations, data over the follow‐up have been obtained via questionnaire surveys and linkage to electronic health records of the UK National Health Service (NHS). The NHS provides most of the healthcare in the country, including inpatient and outpatient care, and record linkage is undertaken using a unique NHS identifier held by all UK residents. At each wave, participants provided informed written consent and research ethics approval was obtained from the NHS London—Harrow Research Ethics Committee (latest reference number 85/0938).

### FFP

Frailty was measured at the clinical examination waves in 2002, 2007, 2012 and 2015 using the FFP, composed of the following five measures, and the thresholds for case definition based on the original study by Fried et al.^4^, ^14^

1. Slow walking speed was defined as when the time spent walking 8 ft was ≥3.73 s for men (women) with height ≤ 173 (≤159) cm and ≥3.20 seconds for men (women) with height >173 (>159) cm.
2. Low grip strength, assessed using a Smedley hand grip dynamometer, was defined for men as ≤29 kg for body mass index (BMI) ≤ 24 kg/m^2^, ≤30 kg for BMI 24.1–28 kg/m^2^ and ≤32 kg for BMI > 28 kg/m^2^. For women, low grip strength was defined as ≤17 kg for BMI ≤ 23 kg/m^2^, ≤17.3 kg for BMI 23.1–26 kg/m^2^, ≤18 kg for BMI 26.1–29 kg/m^2^ and ≤21 kg for BMI > 29 kg/m^2^.
3. Weight loss was defined as unintentional weight loss of 5% or more over the previous year according to Fried's criterion.^4^ Because weight was measured every 5 years, we used a cut‐off of 10% of loss on body weight as used in the Women's Health Aging Study I.^15^
4. Low physical activity was denoted by an energy expenditure of <383 kcal/week for men and <270 kcal/week for women, assessed based on responses to a questionnaire on frequency and duration of participation in 20 physical activities (e.g. cycling, housework and gardening activities). A metabolic equivalent value was assigned to each activity to calculate the energy expenditure of each participant.
5. Exhaustion was defined based on responses to two itemsTrajectories of functioning before onset of frailty extracted from the Center for Epidemiology Studies Depression (CES‐D) scale: ‘I felt that everything I did was an effort in the last week’ and ‘I could not get going in the last week’. If participants answered ‘occasionally or moderate amount of the time (3–4 days)’ or ‘most or all of the time (5–7 days)’ to either of these items, they were categorized as exhausted.

At each of the four waves between 2002 and 2015, the FFP score was calculated as the number of components meeting the criteria described above, resulting in a score ranging from 0 to 5. The poorest performance recorded during this period was used to attribute FFP status to each participant as frail if their score was 3 or more, pre‐frail for a score from 1 to 2 and robust for those with no impaired criteria. Participants classified as pre‐frail and frail were censored at the corresponding date of their worst FFP status and robust participants at last participation (corresponding to their last clinical examination between 2002 and 2015).

### Short‐Form 36 General Health Survey

The Short‐Form 36 General Health Survey (SF‐36) was administered at nine data collection waves (1991, 1995, 1997, 2001, 2002, 2006, 2007, 2012, 2015).^16^, ^17^ The SF‐36 was designed to be a measure of general health status and health‐related quality of life. It contains 36 questions, which consist of eight subscales covering the following domains: physical functioning, bodily pain general health, physical role functioning, vitality, emotional functioning, social role functioning and general mental health. Responses to each question within a dimension were combined to generate eight scores from 0 to 100, with higher scores indicating better health. The SF‐36 was also summarized into physical and mental components scores (PCS and MCS) to measure physical and mental functioning. All subscales contribute in varying proportions to PCS and MCS; these scores range from 0 to 100 and are constructed such that mean scores in the population are 50.

### Covariates

Socio‐demographic variables included age, sex, ethnicity (White or non‐White), current marital status (living with a partner or single) and occupational position at age 50 (high, intermediate and low, reflecting income and status at work).^13^

Health behaviours included smoking status (never smoker, ex‐smoker, current smoker), alcohol consumption (no alcohol in the previous week; moderate, 1–14 units/week; high, >14 units/week), physical activity (less than or at least the recommended 150 min per week of moderate‐to‐vigorous physical activity) and frequency of fruits and vegetables consumption (less than daily, at least once daily).

Body mass index, using height and weight assessed at the clinical examination, was categorized as ≤19.9, 20–24.9, 25–29.9 and ≥30 kg/m^2^.

Chronic conditions were ascertained from clinical examinations in the study and linkage to electronic health records. Three national databases were used: the national Hospital Episode Statistics (HES) database with inpatient and outpatient data; the Mental Health Services Data Set, which in addition to inpatient and outpatient data also has data on care in the community; and the cancer registry. Chronic conditions considered were diabetes (fasting glucose ≥7.0 mmol/L, reported doctor‐diagnosed diabetes, use of diabetes medication, ICD10: E10‐E14), coronary heart disease (12‐lead resting ECG recording, ICD10: I20‐I25), stroke (MONICA‐Augsburg stroke questionnaire, ICD10: I60‐I64), cancer (cancer registry with malignant cancer ICD10: C00;C97), dementia (ICD10: F00‐F03, F05·1, G30, G31), Parkinson's disease (self‐report of longstanding illness, ICD10: G20), chronic obstructive pulmonary disease (self‐report of long‐standing illness, ICD10: J41‐J44), depression (self‐report of long‐standing illness, use of antidepressants, ICD10: F32‐F33) and arthritis (self‐report of long‐standing illness, ICD10: M05, M06, M15‐M19). Multi‐morbidity status was defined as the presence of two or more chronic conditions and was categorized as 0, 1 and 2 or more diseases.

### Mortality

Death from any cause was ascertained using mortality records obtained from the British national mortality register (NHS Central Registry) until October 2019. The tracing exercise was carried out using the National Health Service identification number (NHS‐ID) of each participant.

### Statistical analysis

The association between SF‐36 and FFP status was examined using two approaches: (i) comparison of SF‐36 trajectories over 25 years as a function of FFP status (robust, pre‐frail, frail) of participants and (ii) time‐to‐event analysis to examine the association between poor SF‐36 scores at age 50 and incident frailty, defined using FFP.

#### Trajectory analysis

We compared SF‐36 trajectories (PCS, MCS and the eight subscales, between 1991 and 2015) as a function of FFP status, defined as the poorest score out of four frailty assessments between 2002 and 2015. Trajectories of SF‐36 were estimated using linear mixed models with a backward timescale, anchored to the date of frailty classification such that time 0 was the date at which a participant was classified as their worst FFP‐defined status as being pre‐frail or frail. Data on SF‐36 after frailty/pre‐frailty classification was discarded as our aim was to compare SF‐36 trajectories before the onset of frailty. For participants who remained robust throughout the study, time 0 was the date of clinical examination at last participation. The analysis was adjusted for socio‐demographic factors (sex, ethnicity, marital status and occupation position, age at time 0), frailty status, time terms (time, time^2^ and time^3^) and interactions of time terms with socio‐demographic factors and with FFP status (Model 1); health behaviours (physical activity, alcohol, tobacco and fruits/vegetable consumptions) (Model 2); and BMI and the multi‐morbidity status (Model 3). Besides sex, ethnicity and age at time 0 and FFP status at time 0, data on time varying variables were entered in the analyses concurrent to the measure of SF‐36. Random effects for the intercept and time were included to allow inter‐individual differences in SF‐36 at the intercept (time = 0, at the frailty classification) and in changes in SF‐36 over time (in the rate of change in SF‐36 over the backward follow‐up). Estimates of differences in SF‐36 between FFP status were extracted at time 0, −5, −10, −15, −20 and −25 years from FFP classification.

#### Time‐to‐event analysis

The prospective analyses were based on dichotomous measures of SF‐36 (scores in the worst quartile vs others), retrieved from the wave closest to when participants were 50 (±5) years, to examine associations with FFP‐defined frailty (frail participants compared to robust and pre‐frail participants), over the follow‐up undertaken on participants who were not frail at age 50. These analyses were carried out using an interval‐censored illness–death model with a Weibull distribution to extract hazard ratio (HR) of frailty in those with poor SF‐36 scores compared with others. This method takes interval‐censored nature of the data and competing risk of death into account. Interval censoring was used because measurement of frailty was available only at the waves of data collection and not continuous, such that the exact date of onset could lie in the interval between two clinical examinations. Each SF‐36 scale was analysed separately, and all analyses were first adjusted for socio‐demographics variables (Model 1), then for health behaviours at age 50 (Model 2) and subsequently also for BMI and multi‐morbidity status at age 50 (Model 3).

The analyses were conducted using R software (R Core Team, 2021, version 4.1.2). Linear mixed models, comparisons of SF‐36 trajectories between frail/pre‐frail/robust groups and illness–death models were performed using the *nlme* (version 3.1–153), *emmeans* (version 1.7.2) and *SmoothHazard* (version 1.4.1) packages, respectively. Estimates were reported with 95% confidence intervals (95% CI) and two‐tailed *P*‐values considered significant at 0.05 level.

#### Additional analysis

For the analysis of SF‐36 trajectories, we repeated the main analyses using age as an alternative timescale. Linear mixed‐models were used to examine SF‐36 trajectories between 40 and 85 years according to the worst FFP‐defined frailty status. These analyses were adjusted for the same covariates as in the main analyses and age terms (age, age^2^, age^3^) were used to model the time‐scale of the SF‐36 trajectories. For the time‐to‐event approach, we repeated the analysis using continuous SF‐36 scores; the risk of frailty was estimated for a 5‐point lower SF‐36 score.

## Results

A total of 7044 participants had data on FFP status from clinical examinations (in 2002, 2007, 2012 or 2015) and at least one out of nine measures of SF‐36 between the 1991 and 2015 waves of data collection, constituting the analytic sample of this study (*Figure* S1). Participants included in the analysis were younger than those not in the analysis (49.8 vs 51.9 years; *P* < 0.001, at the first assessment of SF‐36 in 1991), were more likely to be men (29%, 2074/7044 vs 41%, 1339/3264; *P* < 0.001), were Caucasian (92%, 6447/7044 vs 84%, 2734/3264; *P* < 0.001) and had a higher occupational position (42%, 2933/7044 vs 24%, 786/3264; *P* < 0.001) (data not tabulated).

Between 1991 and 2015, over a mean period of 21.4 [standard deviation (SD) 4.0] years, participants provided a mean of 6.4 (SD 2.0) measures of SF‐36; 94% of participants had an SF‐36 measure at the 1991 wave. Over the four measures of FFP between 2002 and 2015, 7% (513/7044) of participants were classified as frail, 64% (4476/7044) as pre‐frail and 29% (2055/7044) as robust based on their worst FFP score over this period. Among those classified as frail and pre‐frail, most (74% for both) participants had not changed their FFP status at the last measurement of FFP (*Table* S1). Given the low proportion of participants who changed FFP status, and the focus of our analyses being on SF‐36 trajectories over 25 years before FFP classification, we did not take these changes into account in the analyses.

*Table* 1 shows that at the 1991 wave, participants who went on to be classified as frail over the follow‐up were more likely to be older (53.1 vs 49.6 and 49.2 years old, *P*‐value < 0.001), were women (46%, 217/468, vs 30%, 1277/4201 and 23%, 445/1928, *P*‐value < 0.001) and were to report at least one chronic disease (10%, 47/468, vs 5%, 218/4201 and 3%, 62/1928, *P*‐value < 0.001) and had lower SF‐36 scores (*P*‐value < 0.001 for all) compared with pre‐frail and robust participants, respectively. Mean age at time 0 (classification of FFP status) was 75.4 (SD 6.3) for frail, 71.8 (SD 6.2) for pre‐frail and 70.7 (SD 5.9) for robust participants.

**Table 1:** Characteristics of participants at the 1991 wave as a function of classification on Fried's frailty phenotype between 2002 and 2015

	Fried's frailty phenotype (FFP)^a^			
	Robust (*N* = 1928)	Pre‐frail (*N* = 4201)	Frail (*N* = 468)	Total (*N* = 6597)
Age, M (SD)	49.2 (5.7)	49.6 (6.0)	53.1 (5.9)	49.7 (6.0)
Range	**39.7–62.7**	**39.6–63.3**	**40.0–62.6**	**39.6–63.3**
Women	445 (23%)	1277 (30%)	217 (46%)	1939 (29%)
White ancestry	1846 (96%)	3821 (91%)	387 (83%)	6054 (92%)
Married/Cohabiting	1632 (85%)	3199 (76%)	299 (64%)	5130 (78%)
High occupational position	929 (48%)	1696 (40%)	121 (26%)	2746 (42%)
Moderate alcohol consumption	1110 (58%)	2335 (56%)	216 (46%)	3661 (56%)
Never smoker	958 (50%)	2171 (52%)	245 (52%)	3374 (51%)
Physical activity at recommended levels	519 (27%)	715 (17%)	39 (8%)	1273 (19%)
Daily fruit and vegetable consumption	366 (19%)	733 (17%)	69 (15%)	1168 (18%)
BMI (kg/m^2^), M (SD)	24.9 (3.1)	25.2 (3.7)	26.6 (4.7)	25.2 (3.6)
Multi‐morbidity status^b^				
0	1866 (97%)	3983 (95%)	421 (90%)	6270 (95%)
1	61 (3%)	206 (5%)	46 (10%)	313 (5%)
2 or more	1 (0%)	12 (0%)	1 (0%)	14 (0%)
SF‐36 component summary scores, M (SD)				
PCS score	53.7 (5.6)	52.3 (7.0)	47.9 (9.7)	52.4 (7.0)
MCS score	52.2 (7.5)	50.6 (8.9)	49.5 (9.7)	51.0 (8.6)
SF‐36 subscales, M (SD)				
Physical functioning	93.3 (10.2)	90.1 (13.7)	80.0 (21.3)	90.3 (13.8)
Physical role	93.0 (20.1)	90.1 (24.3)	79.0 (34.4)	90.1 (24.3)
Bodily pain	85.1 (17.3)	81.4 (20.0)	73.1 (24.2)	81.9 (19.8)
General health	76.4 (15.8)	72.1 (17.7)	66.1 (20.6)	72.9 (17.6)
Social functioning	92.8 (15.4)	89.9 (18.1)	83.2 (23.6)	90.2 (18.0)
Vitality	65.8 (16.5)	60.9 (18.7)	54.5 (20.7)	61.9 (18.5)
Emotional role	91.3 (23.1)	88.0 (26.6)	83.8 (30.5)	88.7 (26.0)
General mental health	78.3 (13.5)	75.3 (15.2)	72.3 (16.9)	76.0 (14.9)

BMI, body mass index; M, mean; MCS, Mental Component Summary score; PCS, Physical Component Summary score; SD, standard deviation; SF‐36, Short‐Form 36 General Health Survey.

Data are N (%) unless stated otherwise.

^a^ FFP classification as robust, pre‐frail and frail was based on the poorest FFP score using four waves of data between 2002 and 2015; robust corresponds to FFP score of 0, pre‐frail to scores of 1 or 2 and frail to scores of 3 or higher.

^b^ The multi‐morbidity status is composed of diabetes, chronic heart disease, stroke, cancer, dementia, Parkinson's disease, chronic obstructive pulmonary disease, depression and arthritis.

### Analysis of SF‐36 trajectories

**Figure 1:**
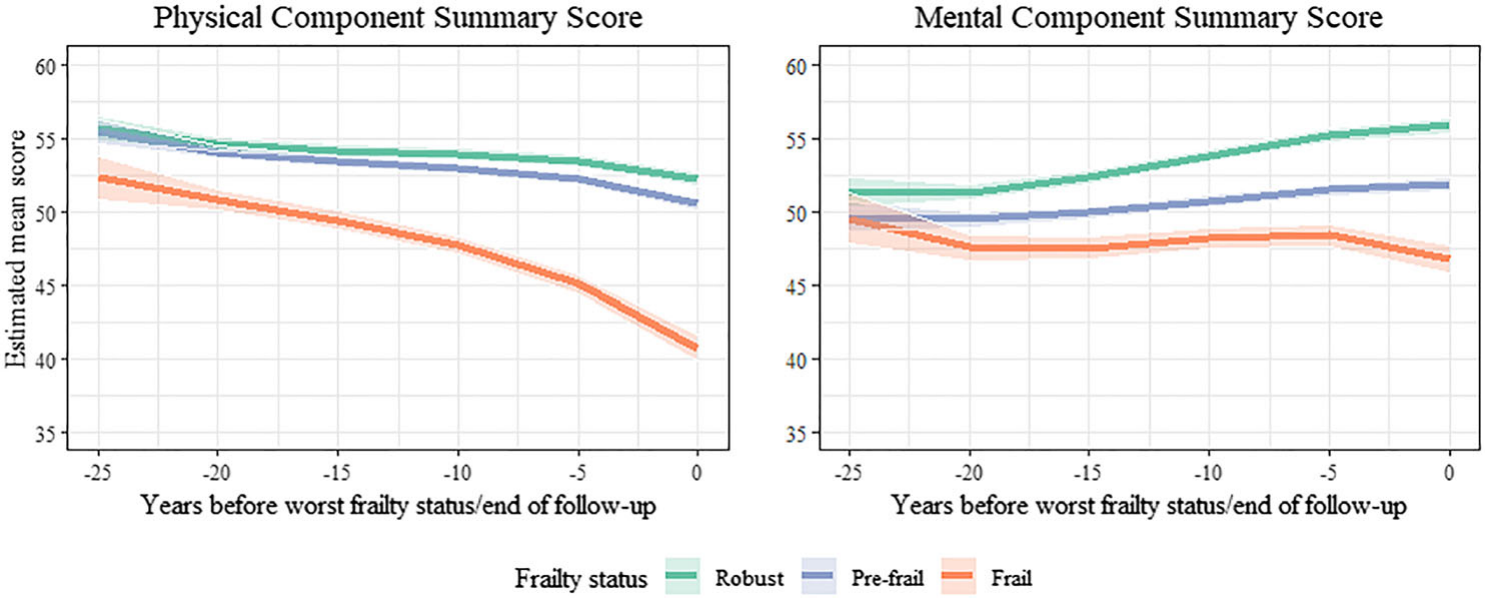
Trajectories of physical and mental component summary scores of the SF‐36 over 25 years using a backward timescale, anchored to classification on Fried's frailty phenotype (FFP).^*^ *Higher SF‐36 scores reflect better health. The backward timescale implies time 0 is the date of FFP classification for prefrail and frail groups and last clinical examination for the robust group. SF‐36 scores were compared over 25 years before time 0. Estimates from linear mixed models; analyses adjusted for time terms (time, time^2^ and time^3^), age at time 0, sex, ethnicity, FFP status, time‐varying covariates (marital status, occupational position, alcohol consumption, smoking status, physical activity, fruit/vegetable consumption, body mass index, multi‐morbidity status) and interaction of time terms with age at time 0, socio‐demographic measures and with FFP status.

*Figure* 1 shows trajectories of SF‐36 component summary scores up to 25 years before onset of pre‐frailty, frailty or end of follow‐up using a backward timescale. Compared with the robust group, frail participants had the poorest scores, particularly in PCS, with an acceleration of the difference at older ages. Estimates of the difference in these scores, every 5 years over the 25 years preceding time 0 are shown in *Table* 2. PCS was lower (3.4, 95% CI 1.6; 5.1) before in frail compared with robust participants 25 years FFP classification (*t* = −25), increasing to a difference of 11.5 (95% CI 10.5; 12.5) at onset of frailty (*t* = 0). Similar differences were observed between frail and pre‐frail participants, at time −25 years (3.1, 95% CI 1.4; 4.8) and increasing at time 0 to 9.8 (95% CI 8.9; 10.7). There were differences in PCS between robust and pre‐frail, starting at time −20 years, but they were smaller, for example, the difference at time 0 was 1.7 (95% CI 1.2; 2.2). *Table* 2 also shows differences in MCS between the robust, pre‐frail and frail groups; the pattern of results was similar to PCS, but the differences between these groups were smaller; at time 0, the difference between the robust and frail was 9.1 (95% CI 8.0; 10.2) and 5.1 (95% CI 4.1; 6.1) between pre‐frail and frail participants.

**Table 2:** Differences in SF‐36 physical and mental component summary scores over 25 years using a backward time‐scale, anchored to classification on Fried's frailty phenotype (FFP)^a^

	Physical component summary score					
	Robust vs pre‐frail		Robust vs frail		Pre‐frail vs frail	
Years preceding poorer frailty status	Difference (95% CI)	*P*	Difference (95% CI)	*P*	Difference (95% CI)	*P*
−25	0.2 (−0.7; 1.2)	0.81	3.4 (1.6; 5.1)	**<0.001**	3.1 (1.4; 4.8)	**<0.001**
−20	0.6 (0.1; 1.0)	**0.009**	3.8 (3.0; 4.7)	**<0.001**	3.3 (2.5; 4.1)	**<0.001**
−15	0.8 (0.4; 1.2)	**<0.001**	4.7 (4.0; 5.5)	**<0.001**	4.0 (3.3; 4.6)	**<0.001**
−10	1.0 (0.6; 1.3)	**<0.001**	6.2 (5.5; 6.9)	**<0.001**	5.2 (4.6; 5.9)	**<0.001**
−5	1.3 (0.8; 1.7)	**<0.001**	8.4 (7.6; 9.2)	**<0.001**	7.2 (6.4; 7.9)	**<0.001**
0	1.7 (1.2; 2.2)	**<0.001**	11.5 (10.5; 12.5)	**<0.001**	9.8 (8.9; 10.7)	**<0.001**

	Mental component summary score					
	Robust vs pre‐frail		Robust vs frail		Pre‐frail vs frail	
Years preceding poorer frailty status	Difference (95% CI)	*P*	Difference (95% CI)	*P*	Difference (95% CI)	*P*
−25	1.7 (0.6; 2.8)	**<0.001**	1.8 (−0.2; 3.8)	0.10	0.1 (−2.0; 2.1)	0.99
−20	1.8 (1.3; 2.4)	**<0.001**	3.8 (2.7; 4.8)	**<0.001**	1.9 (0.9; 2.9)	**<0.001**
−15	2.4 (1.9; 2.9)	**<0.001**	4.8 (3.9; 5.7)	**<0.001**	2.4 (1.6; 3.2)	**<0.001**
−10	3.1 (2.7; 3.6)	**<0.001**	5.6 (4.8; 6.4)	**<0.001**	2.5 (1.7; 3.3)	**<0.001**
−5	3.8 (3.3; 4.2)	**<0.001**	6.8 (5.9; 7.7)	**<0.001**	3.1 (2.2; 3.9)	**<0.001**
0	4.0 (3.4; 4.5)	**<0.001**	9.1 (8.0; 10.2)	**<0.001**	5.1 (4.1; 6.1)	**<0.001**

CI, confidence interval; SF‐36, Short‐Form 36 General Health Survey.

^a^ Higher SF‐36 scores reflect better health. The backward timescale implies time 0 is the date of FFP classification for prefrail and frail groups and last clinical examination for the robust group. SF‐36 scores were compared over 25 years going backward from time 0. Estimates are from linear mixed models; analyses adjusted for time terms (time, time^2^ and time^3^), age at time 0, sex, ethnicity, time‐varying covariates (marital status, occupational position, alcohol consumption, smoking status, physical activity, fruit/vegetable consumption, body mass index, multi‐morbidity status), FFP status and interaction of time terms with age at time 0, socio‐demographic measures and with FFP status.

**Figure 2:**
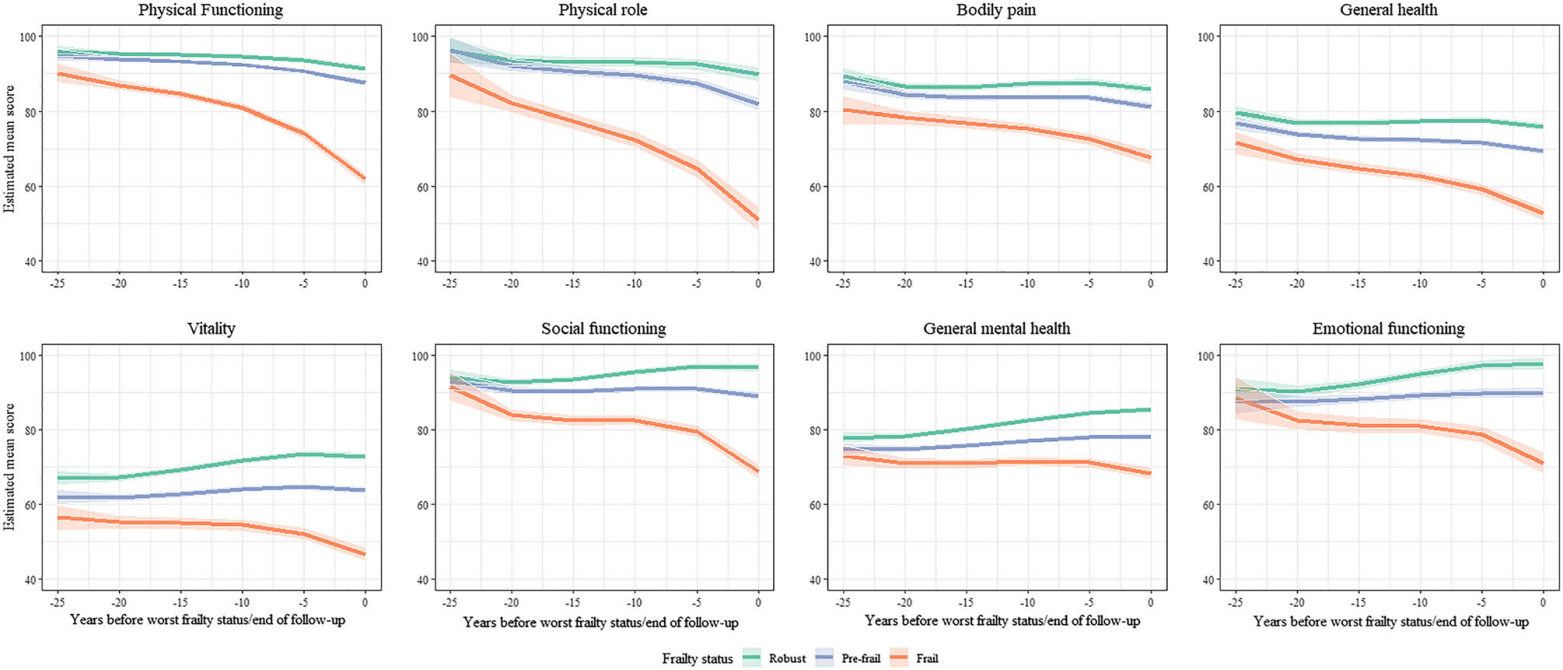
Trajectories of the SF‐36 subscales over 25 years using a backward time‐scale, anchored to classification on Fried's frailty phenotype (FFP).^*^ *Higher SF‐36 scores reflect better health. The backward timescale implies time 0 is the date of FFP classification for prefrail and frail groups and the last clinical examination for the robust group. SF‐36 scores were compared over 25 years before time 0. Estimates from linear mixed models; analyses adjusted for time terms (time, time^2^ and time^3^), age at time 0, sex, ethnicity, FFP status, time‐varying covariates (marital status, occupational position, alcohol consumption, smoking status, physical activity, fruit/vegetable consumption, body mass index, multi‐morbidity status) and interaction of time terms with age at time 0, socio‐demographic measures and with FFP status.

*Figure* 2 shows the scores in SF‐36 subscales (physical functioning, bodily pain, general health, physical role functioning, vitality, emotional functioning, social role functioning and general mental health) from −25 years to time 0 in robust, pre‐frail and frail participants. The estimated differences between robust and pre‐frail (*Table* S2), between robust and frail (*Table* S3) and between pre‐frail and frail (*Table* S4) suggest a similar pattern of findings with significant differences observed 25 years before onset of FFP‐defined frailty.

Results using age as the timescale are shown in *Table* S5 and *Figures* S2 and S3. The largest difference was observed in PCS between robust and frail (21.4, 95% CI 18.5; 24.2) and between the pre‐frail and frail (15.8, 95% CI 13.2; 18.5) groups at age 85. These differences were smaller at younger ages, but PCS scores were higher in robust or pre‐frail compared with frail participants at age 45. The pattern of results for MCS was similar, although like the main results, the differences were smaller in size. The same was observed for subscales of the SF‐36 (*Figure* S3).

### Time‐to‐event analysis

Among the 7044 participants with data on FFP frailty, 5161 (73%) had data on SF‐36 at age 50 (± 5) and were included in the time‐to‐event analyses. Over a mean follow‐up of 21.4 (SD 4.0) years, 269 (5%) of these participants were classified as being frail (three or more impaired FFP criteria). At 50 years, participants who became frail had lower scores compared with non‐frail participants on all SF‐36 subscales and component scores (*Table* S6). *Table* 3 shows results of the association between poor (lowest quartile) SF‐36 scores (PCS, MCS and the eight subscales) at age 50 and FFP frailty over the follow‐up, in analyses that took into account the competing risk of death and interval‐censored nature of the data. In fully adjusted analyses, poor scores on PCS (HR = 2.39, 95% CI 1.85; 3.07) and MCS (HR = 1.49, 95% CI 1.15; 1.93) were associated with higher risk of FFP frailty. Results were similar when SF‐36 scores at age 50 were considered as continuous measures (*Table* S7). A 5‐point lower score in PCS (HR = 1.30, 95% CI 1.22; 1.37) and MCS (HR = 1.14, 95% CI 1.08;1.22) was associated with an increased risk of FFP‐defined frailty.

**Table 3:** Time to event analyses for the prospective associations between poor SF‐36 scores (dichotomous measures) at age 50 and frailty onset (defined using Fried's frailty phenotype) at older ages^a^

	Model 1		Model 2		Model 3	
	HR (95% CI)	*P*‐value	HR (95% CI)	*P*‐value	HR (95% CI)	*P*‐value
SF‐36 component summary scores						
PCS	2.76 (2.14; 3.55)	<0.001	2.55 (1.98; 3.28)	<0.001	2.39 (1.85; 3.07)	<0.001
MCS	1.51 (1.16; 1.95)	0.002	1.53 (1.18; 1.98)	0.001	1.49 (1.15; 1.93)	0.002
SF‐36 subscales						
Physical functioning	2.77 (2.14; 3.60)	<0.001	2.62 (2.02; 3.40)	<0.001	2.36 (1.81; 3.08)	<0.001
Physical role	2.50 (1.95; 3.22)	<0.001	2.46 (1.91; 3.15)	<0.001	2.40 (1.87; 3.09)	<0.001
General health	2.56 (1.99; 3.29)	<0.001	2.33 (1.82; 2.98)	<0.001	2.15 (1.67; 2.76)	<0.001
Body pain	2.11 (1.63; 2.73)	<0.001	2.05 (1.59; 2.64)	<0.001	1.98 (1.53; 2.56)	<0.001
Vitality	2.31 (1.79; 2.96)	<0.001	2.17 (1.69; 2.78)	<0.001	2.12 (1.65; 2.72)	<0.001
Social functioning	2.33 (1.79; 3.03)	<0.001	2.19 (1.68; 2.86)	<0.001	2.07 (1.58; 2.71)	<0.001
Emotional role	1.42 (1.09; 1.86)	0.009	1.46 (1.11; 1.91)	0.006	1.37 (1.04; 1.80)	0.002
Mental health	2.01 (1.55; 2.59)	<0.001	2.01 (1.56; 2.59)	<0.001	1.97 (1.52; 2.55)	<0.001

CI, confidence interval; HR, hazard ratio; MCS, Mental Component Summary score; PCS, Physical Component Summary score; SF‐36, Short‐Form 36 General Health Survey.

Model 1: interval censored illness‐death model with Weibull distribution adjusted for socio‐demographic variables (sex, occupational position, marital status, ethnicity) and wave at age 50, Model 2: models further adjusted for health behaviours at age 50 (alcohol consumption, smoking status, physical activity, fruit/vegetable consumption), Model 3: models further adjusted for body mass index and multi‐morbidity status at age 50.

^a^ Poor scores defined as being in the worst quartile of SF‐36 for each score. Estimates reflect the hazard ratio (HR) of frailty, defined using Fried's Frailty Phenotype, in participants with poor SF‐36 scores (worst quartile) at age 50 compared with all other participants (reference).

## Discussion

Our study using repeated data from nine measures of SF‐36 physical and mental functioning between midlife and old age in individuals who developed FFP‐defined frailty presents three key findings. First, analysis of SF‐36 trajectories showed that participants who developed frailty at older ages had lower SF‐36 scores compared with robust and pre‐frail participants 25 years before the onset of frailty. This finding was confirmed in time‐to‐event analyses where SF‐36 scores at age 50 were associated with a higher risk of incident frailty. Our results suggest that frailty at older ages involves changes over a long period, with heterogeneity in functioning already evident in midlife. The extent to which differences in functioning are evident even earlier remains unknown as we did not have data earlier in the life course. Second, differences in groups defined by frailty status were considerably larger for physical than mental component scores, highlighting the importance of deficits in physical function for FFP frailty. Third, there were only small differences in SF‐36 trajectories between the robust and pre‐frail groups, with considerably larger differences between the pre‐frail and frail groups and between the robust and frail groups.

Heterogeneity in individual trajectories of measures of health and functioning is a hallmark of ageing.^18^, ^19^ The concept of frailty was developed to capture this heterogeneity at older ages in the general population.^1^ Accumulation of three or more of the five components of FFP (slow gait speed, weakness, unintentional weight loss, low physical activity and exhaustion) has been hypothesized to capture a state of vulnerability to risk of adverse health outcomes.^1^ Our study shows that trajectories of physical and mental functioning, as measured by the SF‐36 PCS and MCS, respectively, of individuals who go on to be classified as frail diverged 25 years before the onset of FFP frailty and as early as at age 45 using age as the timescale. Time‐to‐event analyses also showed SF‐36 scores at age 50, 15 years before the recommended age for routine screening of frailty,^2^, ^20^ to be associated with higher risk of FFP‐defined frailty. These findings suggest that there is urgent need to better understand the changes, starting early in the life course, that lead to physical frailty at older ages.

### Strengths and limitations

This study contributes to the emerging literature on determinants and changes leading to FFP frailty. The main strength of the study is modelling the course of changes in SF‐36, a validated and standardized tool,^16^, ^17^ with repeated measurements over a 25‐year period, starting at age 40. The use of Fried's frailty scale,^4^ the most widely used frailty measure in the literature,^7^ is a further strength. A further strength is also the analytic approach, consisting of analysis of trajectories along with time‐to‐event analyses to examine robustness of the findings.

The study findings need to be considered in light of some limitations. First, the measure of FFP frailty was elaborated in 2001 and introduced to the study in 2002; hence, the first of four assessments of FFP in our study was when the mean age of participants was 61. Availability of the measure in 1991 concurrent to the SF‐36 measure would have allowed analyses of onset of frailty earlier in the life course. Second, classifying participants based on their worst FFP score as robust, pre‐frail and frail over four measures does not reflect the dynamics of the frailty process over time. As the focus of our analyses was on changes in functioning, we did not examine changes in the patterns of FFP status using repeated measures. Third, analyses were adjusted for chronic diseases using multi‐morbidity, but examination of changes in SF‐36 trajectories in groups defined by frailty after the occurrence of an acute health event was beyond the scope of the present study. Fourth, whether SF‐36 trajectories as a function of FFP status differ in specific socio‐demographic subgroups could not be examined due to small numbers.

### Comparison with previous studies

A recent meta‐analysis of 22 studies reported poorer quality of life, measured using a range of instruments including the SF‐36, in frail compared with pre‐frail or robust older adults.^21^ Despite methodological heterogeneity, studies that used SF‐36 showed larger differences in physical rather than mental functioning. This was also the case in cross‐sectional analysis of the association between frailty and SF‐36 in the European Male Ageing Study on men between 40 and 79 years.^22^ The concept of frailty is thought to reflect loss of biological reserve, a possible explanation for the stronger association of frailty with physical functioning aspects of the SF‐36.

Prospective studies have examined risk factors for frailty; these include studies on socio‐economic factors,^23^, ^24^ health behaviours,^25^, ^26^, ^27^ obesity,^28^, ^29^ early life adversities^30^, ^31^ and poor self‐rated health in midlife.^32^ One previous study used group‐based modelling on self‐rated health with three assessments over 8 years to show persistent poor self‐rated health to be associated with higher risk of frailty.^33^ The present study adds to the life course approach to frailty using nine measurements of SF‐36 over a 25‐year period to show diverging SF‐36 trajectories as a function of FFP status, both using a backward timescale and age as the timescale. To our knowledge, this is the first study to show robust differences in functioning 25 years before the onset of frailty.

### Meaning of findings

The concept of frailty was designed to capture heterogeneity in health of older adults,^1^ but whether FFP‐defined frailty, as currently measured, is confined to old age is increasingly debated. Landmark studies^9^, ^10^ show frailty to be prevalent in middle‐aged adults in the general population, and like those with frailty at older ages, these individuals have a higher risk of adverse health outcomes. A recent reflection on the use of frailty for clinical practice and public health recommended that the use of a life course approach would provide insight into the development of frailty.^11^ Our findings and previous studies on midlife physical frailty suggest that elaboration of a standard instrument to measure frailty, particularly before age 65, is crucial as current tools were developed for use in older adults. The components of FFP may well be suitable, but the thresholds on each component, defined on an older population in the original study,^4^ may not be ideal before the age 65. We show differences in functioning as early as at age 45 between those who developed frailty later in life and those who remained robust. Whether adapted measures of physical frailty would have picked up frailty earlier in the life course remains unclear.

Differences in SF‐36 scores were observed at age 45 in our study, but whether these differences are clinically meaningful warrants further research.^34^ Studies investigating the minimal clinically important difference in SF‐36 suggest heterogeneous results depending on the target population and methodology.^35^, ^36^ Overall, significant clinical change in SF‐36, at the individual level, appear to be between 5 and 10 points but can range from 2 to 22 points depending on the subscales being considered. In our study, the differences on PCS between robust and frail groups increased from 3.4 at time −25 to 11.5 at time 0. The difference in MCS between these groups was smaller, but there was a fivefold increase between over 25 years. The difference between robust and frail participants in the physical role subscale increased from 6.6 at time −25 to 38.8 at time 0. The additional analyses using age as timescale showed a robust increase in differences in SF‐36 with age, but the point at which these differences become clinically important remains unclear.

## Conclusions

Our analysis shows poorer physical and mental functioning 25 years before the onset of FFP‐defined frailty and as early as age 45 years in those who go on to develop FFP‐defined frailty, particularly in physical functioning. These findings highlight the need for frailty screening prior to old age, perhaps using instruments and thresholds of functional impairment that are better suited to middle‐aged adults for effective prevention.

## Conflict of interest

All authors have completed the ICMJE uniform disclosure form at http://www.icmje.org/coi_disclosure.pdf and declare no support from any organization for the submitted work other than the grants reported in the funding section; no financial relationships with any organizations that might have an interest in the submitted work in the previous 3 years; or no other relationships or activities that could appear to have influenced the submitted work.

## Funding

The Whitehall II study is supported by grants from the National Institute on Aging, NIH (R01AG056477, RF1AG062553); UK Medical Research Council (R024227, S011676); and the Wellcome Trust (221854/Z/20/Z). Séverine Sabia is supported by the French National Research Agency (ANR‐19‐CE36‐0004‐01). Mikaela Bloomberg is supported by the Economic and Social Research Council (ES/P000592/1, https://esrc.ukri.org/). The sponsors had no role in the design and conduct of the study; collection, management, analysis and interpretation of the data; and preparation, review or approval of this manuscript.

## Supporting information


**Table S1.** Changes in frailty status after classification of frailty status using Fried's Frailty Phenotype (FFP)
**Table S2.** Differences in SF‐36 subscales between robust and pre‐frail groups over 25 years using a backward time‐scale, anchored to classification on Fried's Frailty Phenotype (FFP).^*^
**Table S3.** Differences in SF‐36 subscales between robust and frail groups over 25 years using a backward time‐scale, anchored to classification on Fried's Frailty Phenotype (FFP).^*^
**Table S4.** Differences in SF‐36 subscales between pre‐frail and frail groups over 25 years using a backward time‐scale, anchored to classification on Fried's Frailty Phenotype (FFP).^*^
**Table S5.** Differences in SF‐36 physical and mental component summary scores from age 40 to 85 years as a function of classification on Fried's Frailty Phenotype (FFP). *
**Table S6.** Characteristics of participants at age 50 as a function of classification on Fried's Frailty Phenotype (FFP).
**Table S7.** Time to event analyses for the associations between a 5‐point lower score on SF‐36 scores (continuous measure) at age 50 and frailty onset (defined using Fried's Frailty Phenotype) over the follow‐up.^a^
**Figure S1.** Population flow chart.
**Figure S2.** Trajectories of SF‐36 component summary scores from age 40 to 85 years as a function of frailty status defined using Fried's Frailty Phenotype (FFP).*
**Figure S3.** Trajectories of SF‐36 subscales from age 40 to 85 years as a function of frailty status defined using Fried's Frailty Phenotype (FFP). *

## References

[1] Clegg A , Young J , Iliffe S , Rikkert MO , Rockwood K . Frailty in elderly people. Lancet 381:752–762. 23395245 10.1016/S0140-6736(12)62167-9 PMC4098658

[2] Dent E , Lien C , Lim WS , Wong WC , Wong CH , Ng TP , Woo J , Dong B , de la Vega S , Poi PJ , Kamaruzzaman SB . The Asia‐Pacific clinical practice guidelines for the management of frailty. J Am Med Dir Assoc 18:564–575. 28648901 10.1016/j.jamda.2017.04.018

[3] World Health Organization . WHO clinical consortium on healthy ageing. (World Health Organization, 2017).

[4] Fried LP , Tangen CM , Walston J , Newman AB , Hirsch C , Gottdiener J , Seeman T , Tracy R , Kop WJ , Burke G , McBurnie MA . Frailty in older adults: Evidence for a phenotype. J Gerontol A Biol Sci Med Sci 56:M146–M156. 11253156 10.1093/gerona/56.3.m146

[5] Apostolo J , Cooke R , Bobrowicz‐Campos E , Santana S , Marcucci M , Cano A , Vollenbroek‐Hutten M , Germini F , D'Avanzo B , Gwyther H , Holland C . Effectiveness of interventions to prevent pre‐frailty and frailty progression in older adults: A systematic review. JBI Database System Rev Implement Rep 16:140–232. 10.11124/JBISRIR-2017-003382 PMC5771690 29324562

[6] Frost R , Belk C , Jovicic A , Ricciardi F , Kharicha K , Gardner B , Iliffe S , Goodman C , Manthorpe J , Drennan VM , Walters K . Health promotion interventions for community‐dwelling older people with mild or pre‐frailty: A systematic review and meta‐analysis. BMC Geriatr 17:157. 28728570 10.1186/s12877-017-0547-8 PMC5520298

[7] Buta BJ , Walston JD , Godino JG , Park M , Kalyani RR , Xue QL , Bandeen‐Roche K , Varadhan R . Frailty assessment instruments: Systematic characterization of the uses and contexts of highly‐cited instruments. Ageing Res Rev 26:53–61. 26674984 10.1016/j.arr.2015.12.003 PMC4806795

[8] Loecker C , Schmaderer M , Zimmerman L . Frailty in young and middle‐aged adults: An integrative review. J Frailty Aging 10:327–333. 34549246 10.14283/jfa.2021.14

[9] Hanlon P , Nicholl BI , Jani BD , Lee D , McQueenie R , Mair FS . Frailty and pre‐frailty in middle‐aged and older adults and its association with multimorbidity and mortality: A prospective analysis of 493 737 UK Biobank participants. Lancet Public Health 3:e323–e332. 29908859 10.1016/S2468-2667(18)30091-4 PMC6028743

[10] Petermann‐Rocha F , Gray SR , Pell JP , Ho FK , Celis‐Morales C . The joint association of sarcopenia and frailty with incidence and mortality health outcomes: A prospective study. Clin Nutr 40:2427–2434. 10.1016/j.clnu.2020.10.044 33189425

[11] Hoogendijk EO , Afilalo J , Ensrud KE , Kowal P , Onder G , Fried LP . Frailty: implications for clinical practice and public health. Lancet 394:1365–1375. 10.1016/S0140-6736(19)31786-6 31609228

[12] Welstead M , Jenkins ND , Russ TC , Luciano M , Muniz‐Terrera G . A systematic review of frailty trajectories: their shape and influencing factors. Gerontologist 61:e463–e475. 32485739 10.1093/geront/gnaa061 PMC8599181

[13] Marmot MG , Stansfeld S , Patel C , North F , Head J , White I , Brunner E , Feeney A , Smith GD . Health inequalities among British civil servants: The Whitehall II study. Lancet 337:1387–1393. 10.1016/0140-6736(91)93068-k 1674771

[14] Bouillon K , Sabia S , Jokela M , Gale CR , Singh‐Manoux A , Shipley MJ , Kivimäki M , Batty GD . Validating a widely used measure of frailty: Are all sub‐components necessary? Evidence from the Whitehall II cohort study. Age (Dordr) 35:1457–1465. 10.1007/s11357-012-9446-2 PMC3705104 22772579

[15] Boyd CM , Xue QL , Simpson CF , Guralnik JM , Fried LP . Frailty, hospitalization, and progression of disability in a cohort of disabled older women. Am J Med 118:1225–1231. 10.1016/j.amjmed.2005.01.062 16271906

[16] Brazier JE , Harper R , Jones NM , O'cathain A , Thomas KJ , Usherwood T , Westlake L . Validating the SF‐36 health survey questionnaire: New outcome measure for primary care. BMJ 305:160–164. 10.1136/bmj.305.6846.160 PMC1883187 1285753

[17] Walters SJ , Munro JF , Brazier JE . Using the SF‐36 with older adults: A cross‐sectional community‐based survey. Age Ageing 30:337–343. 11509313 10.1093/ageing/30.4.337

[18] Brayne C . The elephant in the room ‐ Healthy brains in later life, epidemiology and public health. Nat Rev Neurosci 8:233–239. 17299455 10.1038/nrn2091

[19] Kuh D , Karunananthan S , Bergman H , Cooper R . A life‐course approach to healthy ageing: Maintaining physical capability. Proc Nutr Soc 73:237–248. 24456831 10.1017/S0029665113003923 PMC3981474

[20] Dent E , Morley JE , Cruz‐Jentoft AJ , Woodhouse L , Rodríguez‐Mañas L , Fried LP , Woo J , Aprahamian I , Sanford A , Lundy J , Landi F . Physical frailty: ICFSR international clinical practice guidelines for identification and management. J Nutr Health Aging 23:771–787. 31641726 10.1007/s12603-019-1273-z PMC6800406

[21] Crocker TF , Brown L , Clegg A , Farley K , Franklin M , Simpkins S , Young J . Quality of life is substantially worse for community‐dwelling older people living with frailty: Systematic review and meta‐analysis. Qual Life Res 28:2041–2056. 10.1007/s11136-019-02149-1 PMC6620381 30875008

[22] O'Connell MD , Tajar A , O'Neill TW , Roberts SA , Lee DM , Pye SR , Silman AJ , Finn JD , Bartfai G , Boonen S , Casanueva FF . Frailty is associated with impaired quality of life and falls in middle‐aged and older European men. J Frailty Aging 2:77–83. 27070662 10.14283/jfa.2013.12

[23] Van der Linden BWA , Cheval B , Sieber S , Orsholits D , Guessous I , Stringhini S , Gabriel R , Aartsen M , Blane D , Courvoisier D , Burton‐Jeangros C . Life course socioeconomic conditions and frailty at older ages. J Gerontol B Psychol Sci Soc Sci 75:1348–1357. 10.1093/geronb/gbz018 PMC7265806 30753721

[24] Dugravot A , Fayosse A , Dumurgier J , Bouillon K , Rayana TB , Schnitzler A , Kivimaki M , Sabia S , Singh‐Manoux A . Social inequalities in multimorbidity, frailty, disability, and transitions to mortality: A 24‐year follow‐up of the Whitehall II cohort study. Lancet Public Health 5:e42–e50. 31837974 10.1016/S2468-2667(19)30226-9 PMC7098476

[25] Savela SL , Koistinen P , Stenholm S , Tilvis RS , Strandberg AY , Pitkälä KH , Salomaa VV , Strandberg TE . Leisure‐time physical activity in midlife is related to old age frailty. J Gerontol A Biol Sci Med Sci 68:1433–1438. 10.1093/gerona/glt029 23525478

[26] Kolehmainen L , Havulinna S , Ngandu T , Strandberg T , Levälahti E , Lehtisalo J , Antikainen R , Hietikko E , Peltonen M , Pölönen A , Soininen H . Earlier life leisure‐time physical activity in relation to age‐related frailty syndrome. Age Ageing 50:161–168. 32808971 10.1093/ageing/afaa132

[27] Strandberg AY , Trygg T , Pitkälä KH , Strandberg TE . Alcohol consumption in midlife and old age and risk of frailty: Alcohol paradox in a 30‐year follow‐up study. Age Ageing 47:248–254. 29088316 10.1093/ageing/afx165

[28] Stenholm S , Strandberg TE , Pitkälä K , Sainio P , Heliövaara M , Koskinen S . Midlife obesity and risk of frailty in old age during a 22‐year follow‐up in men and women: The mini‐Finland follow‐up survey. J Gerontol A Biol Sci Med Sci 69:73–78. 23640762 10.1093/gerona/glt052

[29] Landré B , Czernichow S , Goldberg M , Zins M , Ankri J , Herr M . Association between life‐course obesity and frailty in older adults: Findings in the GAZEL cohort. Obesity (Silver Spring) 28:388–396. 31970909 10.1002/oby.22682

[30] Haapanen MJ , Perälä MM , Osmond C , Salonen MK , Kajantie E , Rantanen T , Simonen M , Pohjolainen P , Eriksson JG , von Bonsdorff MB . Infant and childhood growth and frailty in old age: The Helsinki birth cohort study. Aging Clin Exp Res 31:717–721. 30043315 10.1007/s40520-018-1011-0 PMC6240457

[31] Haapanen MJ , Perälä MM , Salonen MK , Kajantie E , Simonen M , Pohjolainen P , Eriksson JG , von Bonsdorff MB . Early life determinants of frailty in old age: The Helsinki birth cohort study. Age Ageing 47:569–575. 29659671 10.1093/ageing/afy052

[32] Huohvanainen E , Strandberg AY , Stenholm S , Pitkälä KH , Tilvis RS , Strandberg TE . Association of self‐rated health in midlife with mortality and old age frailty: A 26‐year follow‐up of initially healthy men. J Gerontol A Biol Sci Med Sci 71:923–928. 26774116 10.1093/gerona/glv311

[33] Chu WM , Ho HE , Yeh CJ , Hsiao YH , Hsu PS , Lee SH , Lee MC . Self‐rated health trajectory and frailty among community‐dwelling older adults: Evidence from the Taiwan longitudinal study on aging (TLSA). BMJ Open 11:e049795. 10.1136/bmjopen-2021-049795 PMC8351513 34362805

[34] Weinfurt KP . Clarifying the meaning of clinically meaningful benefit in clinical research: Noticeable change vs valuable change. JAMA 322:2381–2382. 10.1001/jama.2019.18496 31790549

[35] Jayadevappa R , Cook R , Chhatre S . Minimal important difference to infer changes in health‐related quality of life‐A systematic review. J Clin Epidemiol 89:188–198. 28676426 10.1016/j.jclinepi.2017.06.009

[36] Mouelhi Y , Jouve E , Castelli C , Gentile S . How is the minimal clinically important difference established in health‐related quality of life instruments? Review of anchors and methods. Health Qual Life Outcomes 18:136. 32398083 10.1186/s12955-020-01344-w PMC7218583

[37] von Haehling S , Coats AJS , Anker SD . Ethical guidelines for publishing in the Journal of Cachexia, Sarcopenia and Muscle: Update. J Cachexia Sarcopenia Muscle 2021;12:2259–2261. 34904399 10.1002/jcsm.12899 PMC8718061

