# Supplementary material for: Trajectories of physical and mental functioning over 25 years before onset of frailty: results from the Whitehall II cohort study
Source: J Cachexia Sarcopenia Muscle. 2022 Nov 17;14(1):288–97. doi: 10.1002/jcsm.13129 (PMC9891967; doi:10.1002/jcsm.13129)
Supplement: Supplementary file 1 — Table S1. Changes in frailty status after classification of frailty status using Fried's Frailty Phenotype (FFP) Table S2. Differences in SF‐36 subscales between robust and pre‐frail groups over 25 years using a backward time‐scale, anchored to classification on Fried's Frailty Phenotype (FFP).* Table S3. Differences in SF‐36 subscales between robust and frail groups over 25 years using a backward time‐scale, anchored to classification on Fried's Frailty Phenotype (FFP).* Table S4. Differences in SF‐36 subscales between pre‐frail and frail groups over 25 years using a backward time‐scale, anchored to classification on Fried's Frailty Phenotype (FFP).* Table S5. Differences in SF‐36 physical and mental component summary scores from age 40 to 85 years as a function of classification on Fried's Frailty Phenotype (FFP). * Table S6. Characteristics of participants at age 50 as a function of classification on Fried's Frailty Phenotype (FFP). Table S7. Time to event analyses for the associations between a 5‐point lower score on SF‐36 scores (continuous measure) at age 50 and frailty onset (defined using Fried's Frailty Phenotype) over the follow‐up.a Figure S1. Population flow chart. Figure S2. Trajectories of SF‐36 component summary scores from age 40 to 85 years as a function of frailty status defined using Fried's Frailty Phenotype (FFP).* Figure S3. Trajectories of SF‐36 subscales from age 40 to 85 years as a function of frailty status defined using Fried's Frailty Phenotype (FFP). * [file JCSM-14-288-s001.docx]

**Trajectories of physical and mental functioning over 25 years before onset of frailty: Results from the Whitehall II cohort study.**

Benjamin Landré,^1,*^ Céline Ben Hassen,^1^ Mika Kivimaki,^1,2^ Mikaela Bloomberg,^2^ Aline Dugravot,^1^ Alexis Schniztler,^1^ Séverine Sabia,^1,2^ Archana Singh-Manoux^1,2^

^1^Université Paris Cité, Inserm U1153, CRESS, Epidemiology of Ageing and Neurodegenerative diseases, Paris, France

^2^Department of Epidemiology and Public Health, University College London, UK

*Corresponding author & address

Benjamin Landré

Université Paris Cité

Inserm U1153, EpiAgeing

10 avenue de Verdun, 75010 Paris, France

Email : [benjamin.landre@inserm.fr](mailto:benjamin.landre@inserm.fr)

ORCID : 0000-0002-3893-4197

Twitter : @epiageing

**Supplementary material**

**Table S1. Changes in frailty status after classification of frailty status using Fried’s Frailty Phenotype (FFP)**

|  | | **Classification in the analysis**  **FRAIL^a^**  **(N = 513)** | **Classification in the analysis**  **PRE-FRAIL^a^**  **(N = 4476)** |
| --- | --- | --- | --- |
| **Classification at last measure** | | **N(%)** | **N(%)** |
|  | **Robust** | **17 (3.3)** | **1168 (26.1)** |
|  | **Pre-frail** | **118 (23.0)** | **3308 (73.9)** |
|  | **Frail** | **378 (73.7)** | **NA** |

**^a^****The poorest FFP score using data from four waves between 2002 and 2015 was used to define frailty status; robust corresponds to a score of 0, pre-frail to scores of 1 or 2, and frail to scores of 3 or higher.**

**Table S2. Differences in SF-36 subscales between robust and pre-frail groups over 25 years using a backward time-scale, anchored to classification on Fried’s Frailty Phenotype (FFP). ^*^**

|  | **Physical functioning** | |  | **Physical role** | |  | **Bodily pain** | |  | **General health** | |
| --- | --- | --- | --- | --- | --- | --- | --- | --- | --- | --- | --- |
| **Year** | **Difference (95%CI)** | **P** |  | **Difference (95%CI)** | **P** |  | **Difference (95%CI)** | **P** |  | **Difference (95%CI)** | **P** |
| **-25** | 1.2 (-0.5; 2.9) | 0.218 |  | -0.2 (-4.0; 3.6) | 0.99 |  | 1.6 (-0.9; 4.1) | 0.28 |  | 2.8 (0.8; 4.8) | 0.003 |
| **-20** | 1.4 (0.6; 2.3) | <0.001 |  | 1.5 (0.0; 3.0) | 0.06 |  | 2.2 (1.0; 3.4) | <0.001 |  | 3.1 (1.9; 4.3) | <0.001 |
| **-15** | 1.8 (1.1; 2.5) | <0.001 |  | 2.5 (1.2; 3.9) | <0.001 |  | 2.8 (1.8; 3.9) | <0.001 |  | 4.0 (3.0; 5.0) | <0.001 |
| **-10** | 2.3 (1.6; 3.0) | <0.001 |  | 3.5 (2.2; 4.8) | <0.001 |  | 3.5 (2.5; 4.4) | <0.001 |  | 5.2 (4.2; 6.1) | <0.001 |
| **-5** | 3.0 (2.1; 3.8) | <0.001 |  | 5.1 (3.6; 6.6) | <0.001 |  | 4.1 (3.1; 5.1) | <0.001 |  | 6.1 (5.1; 7.1) | <0.001 |
| **0** | 3.8 (2.8; 4.9) | <0.001 |  | 7.9 (5.9; 9.9) | <0.001 |  | 4.7 (3.5; 6.0) | <0.001 |  | 6.5 (5.3; 7.6) | <0.001 |
|  |  | | | | | | | | | | |
|  | **Vitality** | |  | **Social functioning** | |  | **General mental health** | |  | **Emotional role** | |
| **-25** | 5.1 (2.9; 7.2) | <0.001 |  | 1.1 (-1.3; 3.5) | 0.535 |  | 2.9 (1.2; 4.7) | <0.001 |  | 3.3 (-0.3; 6.9) | 0.082 |
| **-20** | 5.4 (4.2; 6.6) | <0.001 |  | 2.1 (1.0; 3.2) | <0.001 |  | 3.5 (2.5; 4.4) | <0.001 |  | 2.7 (1.1; 4.3) | <0.001 |
| **-15** | 6.5 (5.5; 7.5) | <0.001 |  | 3.3 (2.3; 4.2) | <0.001 |  | 4.4 (3.6; 5.3) | <0.001 |  | 3.9 (2.5; 5.3) | <0.001 |
| **-10** | 7.8 (6.8; 8.8) | <0.001 |  | 4.6 (3.7; 5.4) | <0.001 |  | 5.5 (4.7; 6.3) | <0.001 |  | 5.8 (4.6; 7.0) | <0.001 |
| **-5** | 8.8 (7.8; 9.8) | <0.001 |  | 6.0 (5.1; 7.0) | <0.001 |  | 6.5 (5.7; 7.3) | <0.001 |  | 7.5 (6.1; 8.8) | <0.001 |
| **0** | 9.1 (7.9; 10.3) | <0.001 |  | 7.6 (6.3; 8.8) | <0.001 |  | 7.2 (6.2; 8.2) | <0.001 |  | 7.7 (6.0; 9.4) | <0.001 |

Abbreviations: SF-36, Short Form 36 General Health Survey; CI, Confidence Interval.
* Higher SF-36 scores reflect better health. The backward **time-scale implies** time 0 **is** the date of **FFP** classification **for prefrail and frail groups and last clinical examination for the robust group.** SF-36 scores were compared over 25 years **going backward from** time 0. Estimates **are** from linear mixed models; analyses adjusted for time terms (time, time ² and time^3^), age at **time 0,** sex, ethnicity**, time-varying covariates (**marital status, occupational position, alcohol consumption, smoking status, physical activity, fruit/vegetable consumption, body mass index, multimorbidity status**), FFP** status, and interaction of time terms with **age at time 0,** socio demographic measures and with **FFP** status.

**Table S3. Differences in SF-36 subscales between robust and frail groups** **over 25 years using a backward time-scale, anchored to classification on Fried’s Frailty Phenotype (FFP).^*^**

|  | **Physical functioning** | |  | **Physical role** | |  | **Bodily pain** | |  | **General health** | |
| --- | --- | --- | --- | --- | --- | --- | --- | --- | --- | --- | --- |
| **Year** | **Difference (95%CI)** | **P** |  | **Difference (95%CI)** | **P** |  | **Difference (95%CI)** | **P** |  | **Difference (95%CI)** | **P** |
| **-25** | 5.9 (2.7; 9.1) | < 0.001 |  | 6.6 (-0.4; 13.7) | 0.07 |  | 9.2 (4.5; 13.8) | <0.001 |  | 8.1 (4.3; 11.8) | <0.001 |
| **-20** | 8.3 (6.8; 9.8) | < 0.001 |  | 11.7 (8.9; 14.5) | <0.001 |  | 8.2 (6.1; 10.4) | <0.001 |  | 9.8 (7.6; 11.9) | <0.001 |
| **-15** | 10.5 (9.1; 11.8) | < 0.001 |  | 15.9 (13.4; 18.4) | <0.001 |  | 9.4 (7.5; 11.3) | <0.001 |  | 12.0 (10.1; 13.9) | <0.001 |
| **-10** | 13.8 (12.4; 15.1) | < 0.001 |  | 20.8 (18.3; 23.2) | <0.001 |  | 12.0 (10.2; 13.8) | <0.001 |  | 14.9 (13.1; 16.7) | <0.001 |
| **-5** | 19.6 (18.1; 21.2) | < 0.001 |  | 27.9 (24.9; 30.8) | <0.001 |  | 15.2 (13.2; 17.1) | <0.001 |  | 18.6 (16.7; 20.5) | <0.001 |
| **0** | 29.6 (27.6; 31.5) | < 0.001 |  | 38.8 (34.9; 42.7) | <0.001 |  | 18.2 (15.8; 20.6) | <0.001 |  | 23.4 (21.2; 25.6) | <0.001 |
|  |  | | | | | | | | | | |
|  | **Vitality** | |  | **Social functioning** | |  | **General mental health** | |  | **Emotional role** | |
| **-25** | 10.6 (6.6; 14.5) | <0.001 |  | 2.5 (-2.1; 7.1) | 0.419 |  | 4.8 (1.5; 8.1) | 0.002 |  | 2.5 (-4.3; 9.2) | 0.67 |
| **-20** | 12.0 (9.8; 14.2) | <0.001 |  | 8.8 (6.8; 10.8) | <0.001 |  | 7.4 (5.6; 9.1) | <0.001 |  | 7.7 (4.8; 10.6) | <0.001 |
| **-15** | 14.3 (12.3; 16.2) | <0.001 |  | 11.2 (9.4; 12.9) | <0.001 |  | 9.3 (7.7; 10.8) | <0.001 |  | 10.9 (8.4; 13.4) | <0.001 |
| **-10** | 17.4 (15.6; 19.2) | <0.001 |  | 12.9 (11.3; 14.5) | <0.001 |  | 11.1 (9.6; 12.5) | <0.001 |  | 13.8 (11.5; 16.2) | <0.001 |
| **-5** | 21.3 (19.4; 23.3) | <0.001 |  | 17.3 (15.5; 19.2) | <0.001 |  | 13.4 (11.9; 15.0) | <0.001 |  | 18.5 (15.9; 21.1) | <0.001 |
| **0** | 26.2 (23.9; 28.4) | <0.001 |  | 27.8 (25.4; 30.2) | <0.001 |  | 17.1 (15.2; 18.9) | <0.001 |  | 26.6 (23.2; 30.0) | <0.001 |

Abbreviations: SF-36, Short Form 36 General Health Survey; CI, Confidence Interval.
* Higher SF-36 scores reflect better health. The backward time-scale implies time 0 is the date of FFP classification **for prefrail and frail groups and last clinical examination for the robust group.** SF-36 scores were compared over 25 years **going backward from** time 0. Estimates **are** from linear mixed models; analyses adjusted for time terms (time, time ² and time^3^), age at **time 0**, sex, ethnicity**, time-varying covariates (**marital status, occupational position, alcohol consumption, smoking status, physical activity, fruit/vegetable consumption, body mass index, multimorbidity status**), FFP** status, and interaction of time terms with **age at time 0,** socio demographic measures and with **FFP** status.

**Table S4. Differences in SF-36 subscales between pre-frail and frail groups** **over 25 years using a backward time-scale, anchored to classification on Fried’s Frailty Phenotype (FFP). ^*^**

|  | **Physical functioning** | |  | **Physical role** | |  | **Bodily pain** | |  | **General health** | |
| --- | --- | --- | --- | --- | --- | --- | --- | --- | --- | --- | --- |
| **Year** | **Difference (95%CI)** | **P** |  | **Difference (95%CI)** | **P** |  | **Difference (95%CI)** | **P** |  | **Difference (95%CI)** | **P** |
| **-25** | 4.7 (1.5; 7.9) | 0.002 |  | 6.9 (-0.3; 14.0) | 0.06 |  | 7.5 (2.9; 12.2) | <0.001 |  | 5.3 (1.6; 9.0) | 0.002 |
| **-20** | 6.9 (5.4; 8.3) | <0.001 |  | 10.2 (7.5; 12.9) | <0.001 |  | 6.0 (4.0; 8.1) | <0.001 |  | 6.7 (4.7; 8.7) | <0.001 |
| **-15** | 8.7 (7.4; 9.9) | <0.001 |  | 13.4 (11.0; 15.7) | <0.001 |  | 6.6 (4.8; 8.4) | <0.001 |  | 8.0 (6.2; 9.8) | <0.001 |
| **-10** | 11.5 (10.2; 12.7) | <0.001 |  | 17.3 (14.9; 19.6) | <0.001 |  | 8.5 (6.8; 10.2) | <0.001 |  | 9.7 (8.0; 11.4) | <0.001 |
| **-5** | 16.7 (15.2; 18.2) | <0.001 |  | 22.8 (20.0; 25.5) | <0.001 |  | 11.0 (9.2; 12.9) | <0.001 |  | 12.5 (10.7; 14.3) | <0.001 |
| **0** | 25.7 (23.9; 27.6) | <0.001 |  | 30.9 (27.3; 34.5) | <0.001 |  | 13.5 (11.2; 15.7) | <0.001 |  | 16.9 (14.8; 19.0) | <0.001 |
|  |  | | | | | | | | | | |
|  | **Vitality** | |  | **Social functioning** | |  | **General mental health** | |  | **Emotional role** | |
| **-25** | 5.5 (1.6; 9.5) | 0.003 |  | 1.4 (-3.2; 6.0) | 0.769 |  | 1.9 (-1.4; 5.2) | 0.383 |  | -0.8 (-7.6; 6.0) | 0.96 |
| **-20** | 6.6 (4.5; 8.7) | <0.001 |  | 6.7 (4.8; 8.6) | <0.001 |  | 3.9 (2.2; 5.6) | <0.001 |  | 5.0 (2.2; 7.8) | <0.001 |
| **-15** | 7.8 (6.0; 9.6) | <0.001 |  | 7.9 (6.3; 9.5) | <0.001 |  | 4.8 (3.4; 6.3) | <0.001 |  | 7.0 (4.6; 9.3) | <0.001 |
| **-10** | 9.6 (7.9; 11.3) | <0.001 |  | 8.3 (6.8; 9.8) | <0.001 |  | 5.5 (4.2; 6.9) | <0.001 |  | 8.0 (5.9; 10.2) | <0.001 |
| **-5** | 12.5 (10.7; 14.3) | <0.001 |  | 11.3 (9.6; 13.0) | <0.001 |  | 6.9 (5.4; 8.4) | <0.001 |  | 11.0 (8.6; 13.4) | < 0.001 |
| **0** | 17.1 (15.0; 19.1) | <0.001 |  | 20.2 (18.0; 22.4) | <0.001 |  | 9.8 (8.1; 11.6) | <0.001 |  | 18.9 (15.7; 22.0) | <0.001 |

Abbreviations: SF-36, Short Form 36 General Health Survey; CI, Confidence Interval.
Higher SF-36 scores reflect better health. The backward **time-scale implies** time 0 **is** the date of **FFP** classification for prefrail and frail **groups and last clinical examination for the robust group.** SF-36 scores were compared over 25 years **going backward from** time 0. Estimates **are** from linear mixed models; analyses adjusted for time terms (time, time ² and time^3^), age at **time 0**, sex, ethnicity**, time-varying covariates** (marital status, occupational position, alcohol consumption, smoking status, physical activity, fruit/vegetable consumption, body mass index, multimorbidity status**), FFP** status, and interaction of time terms with **age at time 0,** socio demographic measures and with **FFP** status.

**Table S5. Differences in SF-36 physical and mental component summary scores from age 40 to 85 years as a function of classification on Fried’s Frailty Phenotype (FFP). ***

|  | **Physical component summary score** | | | | | | | | |
| --- | --- | --- | --- | --- | --- | --- | --- | --- | --- |
|  | **Robust vs. Pre-frail** | |  | **Robust vs. Frail** | |  | **Pre-frail vs. Frail** | |  |
| **Age** | **Difference**  **(95%CI)** | **P** |  | **Difference**  **(95%CI)** | **P** |  | **Difference**  **(95%CI)** | **P** |  |
| **40** | 0.7 (-0.3; 1.7) | 0.24 |  | 1.6 (-0.8; 4.0) | 0.25 |  | 0.9 (-1.4; 3.2) | 0.62 |  |
| **45** | 0.9 (0.4; 1.5) | <0.001 |  | 3.1 (1.9; 4.4) | <0.001 |  | 2.2 (1.0; 3.4) | <0.001 |  |
| **50** | 1.2 (0.8; 1.6) | <0.001 |  | 4.2 (3.4; 5.1) | <0.001 |  | 3.0 (2.2; 3.9) | <0.001 |  |
| **55** | 1.5 (1.1; 1.8) | <0.001 |  | 5.1 (4.4; 5.9) | <0.001 |  | 3.7 (3.0; 4.4) | <0.001 |  |
| **60** | 1.8 (1.4; 2.2) | <0.001 |  | 6.1 (5.4; 6.8) | <0.001 |  | 4.3 (3.6; 5.0) | <0.001 |  |
| **65** | 2.2 (1.8; 2.7) | <0.001 |  | 7.4 (6.6; 8.2) | <0.001 |  | 5.2 (4.4; 5.9) | <0.001 |  |
| **70** | 2.8 (2.2; 3.3) | <0.001 |  | 9.3 (8.4; 10.3) | <0.001 |  | 6.5 (5.7; 7.4) | <0.001 |  |
| **75** | 3.5 (2.8; 4.2) | <0.001 |  | 12.1 (11.0; 13.3) | <0.001 |  | 8.6 (7.5; 9.7) | <0.001 |  |
| **80** | 4.4 (3.2; 5.6) | <0.001 |  | 16.0 (14.4; 17.7) | <0.001 |  | 11.6 (10.1; 13.1) | <0.001 |  |
| **85** | 5.5 (3.4; 7.6) | <0.001 |  | 21.4 (18.5; 24.2) | <0.001 |  | 15.8 (13.2; 18.5) | <0.001 |  |
|  | **Mental component summary score** | | | | | | | | |
|  | **Robust vs. Pre-frail** | |  | **Robust vs. Frail** | |  | **Pre-frail vs. Frail** | |  |
| **Age** | **Difference**  **(95%CI)** | **P** |  | **Difference**  **(95%CI)** | **P** |  | **Difference**  **(95%CI)** | **P** |  |
| **40** | 0.7 (-0.5; 1.9) | 0.39 |  | 0.7 (-2.1; 3.6) | 0.81 |  | 0.1 (-2.6; 2.8) | 0.99 |  |
| **45** | 1.4 (0.8; 2.1) | <0.001 |  | 2.4 (0.9; 4.0) | <0.001 |  | 1.0 (-0.5; 2.5) | 0.25 |  |
| **50** | 1.9 (1.4; 2.4) | <0.001 |  | 3.7 (2.6; 4.8) | <0.001 |  | 1.8 (0.8; 2.8) | <0.001 |  |
| **55** | 2.2 (1.7; 2.6) | <0.001 |  | 4.7 (3.8; 5.6) | <0.001 |  | 2.5 (1.7; 3.3) | <0.001 |  |
| **60** | 2.3 (1.9; 2.7) | <0.001 |  | 5.4 (4.6; 6.2) | <0.001 |  | 3.1 (2.4; 3.9) | <0.001 |  |
| **65** | 2.4 (1.9; 2.8) | <0.001 |  | 6.1 (5.3; 6.9) | <0.001 |  | 3.7 (2.9; 4.4) | <0.001 |  |
| **70** | 2.5 (2.0; 3.0) | <0.001 |  | 6.7 (5.8; 7.6) | <0.001 |  | 4.2 (3.4; 5.0) | <0.001 |  |
| **75** | 2.8 (2.0; 3.5) | <0.001 |  | 7.4 (6.4; 8.5) | <0.001 |  | 4.7 (3.7; 5.7) | <0.001 |  |
| **80** | 3.2 (1.9; 4.4) | <0.001 |  | 8.4 (6.7; 10.1) | <0.001 |  | 5.2 (3.7; 6.8) | <0.001 |  |
| **85** | 3.9 (1.6; 6.2) | <0.001 |  | 9.7 (6.5; 12.8) | <0.001 |  | 5.8 (2.9; 8.6) | <0.001 |  |

Abbreviations: SF-36, Short Form 36 General Health Survey; CI, Confidence Interval.

**^*^** Higher SF-36 scores reflect better health. Estimates from linear mixed models; analyses included age terms (age, age², and age^3^), sex, ethnicity, **time-varying covariates (**marital status, **occupational** position, alcohol consumption, smoking status, physical activity, fruit/vegetable consumption, body mass index, multimorbidity **score), FFP** status, and interaction of age terms with socio demographic measures and with **FFP** status.

**Table S6. Characteristics of participants at age 50 as a function of classification on Fried’s Frailty Phenotype (FFP).**

|  | **Fried’s Frailty Phenotype** | |  |
| --- | --- | --- | --- |
|  | **Robust or pre-frail**  **(N=4 892)** | **Frail**  **(N=269)** | **Total**  **(N=5 161)** |
| Age, M (SD) | 50.6 (1.7) | 51.2 (3) | 50.6 (2) |
| Women | 1325 (27%) | 129 (48%) | 1454 (28%) |
| White | 4543 (93%) | 217 (81%) | 4760 (92%) |
| Married/Cohabiting | 3863 (79%) | 159 (59%) | 4022 (78%) |
| High **occupational** position | 2160 (44%) | 62 (23%) | 2222 (43%) |
| Moderate alcohol consumption | 2491 (51%) | 107 (40%) | 2598 (50%) |
| Never smoker | 2463 (50%) | 142 (53%) | 2605 (51%) |
| Physical activity at recommended levels | 1482 (30%) | 28 (10%) | 1510 (29%) |
| Daily fruit & vegetable consumption | 1423 (29%) | 64 (24%) | 1487 (29%) |
| **BMI (kg/m²), M (SD)** | 25.6 (4.0) | 27.3 (5.0) | 25.7 (4) |
| **Multimorbidity status^a^** |  |  |  |
| 0 | 4478 (92%) | 234 (87%) | 4712 (91%) |
| 1 | 393 (8%) | 32 (12%) | 425 (8%) |
| 2 or more | 21 (0%) | 3 (1%) | 24 (1%) |
| **SF-36 component summary scores, M (SD)** |  |  |  |
| PCS | 52.3 (7.0) | 46.2 (11.1) | 52.0 (7.4) |
| MCS | 49.8 (9.5) | 46.5 (11.5) | 49.6 (9.7) |
| **SF-36 subscales, M (SD)** |  |  |  |
| Physical functioning scale | 90.6 (12.3) | 76.8 (23.9) | 89.8 (14.1) |
| Physical role scale | 88.6 (26.4) | 71.9 (38.5) | 87.8 (27.4) |
| Bodily pain scale | 81.6 (19.7) | 68.9 (24.9) | 80.9 (20.2) |
| General health scale | 70.8 (18.5) | 60.2 (22.7) | 70.3 (18.9) |
| Social functioning scale | 88.3 (19.3) | 76.0 (27.5) | 87.6 (20.0) |
| Vitality scale | 60.1 (19.1) | 47.5 (21.9) | 59.5 (19.5) |
| Emotional role scale | 86.0 (29.1) | 77.3 (36.7) | 85.5 (29.6) |
| General mental health scale | 74.1 (15.9) | 67.3 (18.9) | 73.8 (16.1) |

Abbreviations: M, mean; SD, standard deviation; BMI, Body Mass Index; SF-36, Short Form 36 General Health Survey; PCS, Physical Component Summary score; MCS, Mental Component Summary score.

Data are N (%) unless stated otherwise.

**^a^The** multimorbidity status is composed of diabetes, chronic heart disease, stroke, cancer, dementia, Parkinson’s disease, chronic obstructive pulmonary disease, depression, and arthritis.

**Table S7. Time to event analyses for the associations between a 5-point lower score on SF-36 scores (continuous measure) at age 50 and frailty onset (defined using Fried’s Frailty Phenotype) over the follow-up.^a^**

|  | **Model 1** | | **Model 2** | | **Model 3** | |
| --- | --- | --- | --- | --- | --- | --- |
|  | HR (95%CI) | P-value | HR (95%CI) | P-value | HR (95%CI) | P-value |
| **SF-36 component summary scores** |  |  |  |  |  |  |
| PCS | 1.33 (1.27; 1.43) | < 0.001 | 1.32 (1.23; 1.39) | < 0.001 | 1.30 (1.22; 1.37) | < 0.001 |
| MCS | 1.15 (1.12; 1.18) | < 0.001 | 1.15 (1.09; 1.22) | < 0.001 | 1.14 (1.08; 1.22) | < 0.001 |
| **SF-36 subscales** |  |  |  |  |  |  |
| Physical functioning | 1.06 (1.04; 1.08) | < 0.001 | 1.15 (1.12; 1.18) | < 0.001 | 1.14 (1.11; 1.18) | < 0.001 |
| Physical role | 1.14 (1.10; 1.18) | < 0.001 | 1.06 (1.04; 1.08) | < 0.001 | 1.05 (1.04; 1.08) | < 0.001 |
| General Health | 1.10 (1.08; 1.14) | < 0.001 | 1.12 (1.09; 1.16) | < 0.001 | 1.11 (1.09; 1.15) | < 0.001 |
| Body pain | 1.15 (1.09; 1.20) | < 0.001 | 1.10 (1.06; 1.12) | < 0.001 | 1.09 (1.06; 1.12) | < 0.001 |
| Vitality | 1.14 (1.11; 1.18) | < 0.001 | 1.14 (1.10; 1.18) | < 0.001 | 1.14 (1.10; 1.16) | < 0.001 |
| Social functioning | 1.11 (1.08; 1.12) | < 0.001 | 1.09 (1.06; 1.11) | < 0.001 | 1.09 (1.06; 1.11) | < 0.001 |
| Emotional role | 1.03 (1.01; 1.05) | 0.001 | 1.03 (1.01; 1.05) | < 0.001 | 1.03 (1.01; 1.05) | 0.002 |
| Mental health | 1.11 (1.08; 1.15) | < 0.001 | 1.11 (1.08; 1.15) | < 0.001 | 1.11 (1.08; 1.15) | < 0.001 |

Abbreviations: SF-36**,** Short Form 36 General Health Survey; HR, Hazard Ratio; CI, Confidence Interval; PCS, Physical Component Summary score; MCS, Mental Component Summary score.

^a^ Estimates reflect the Hazard Ratio (HR) of incident frailty for a 5-point lower SF-36 score at age 50.

Model 1: interval censoring illness-death model with Weibull distribution adjusted for socio-demographic variables (sex, **occupational** position, marital status, ethnicity) and wave at age 50, Model 2: models further adjusted for health behaviours at age 50 (alcohol consumption, smoking status, physical activity, fruit/vegetable consumption), Model 3: models further adjusted for body mass index and multimorbidity status at age 50.

**Fig. S1. Population flow chart.**

**
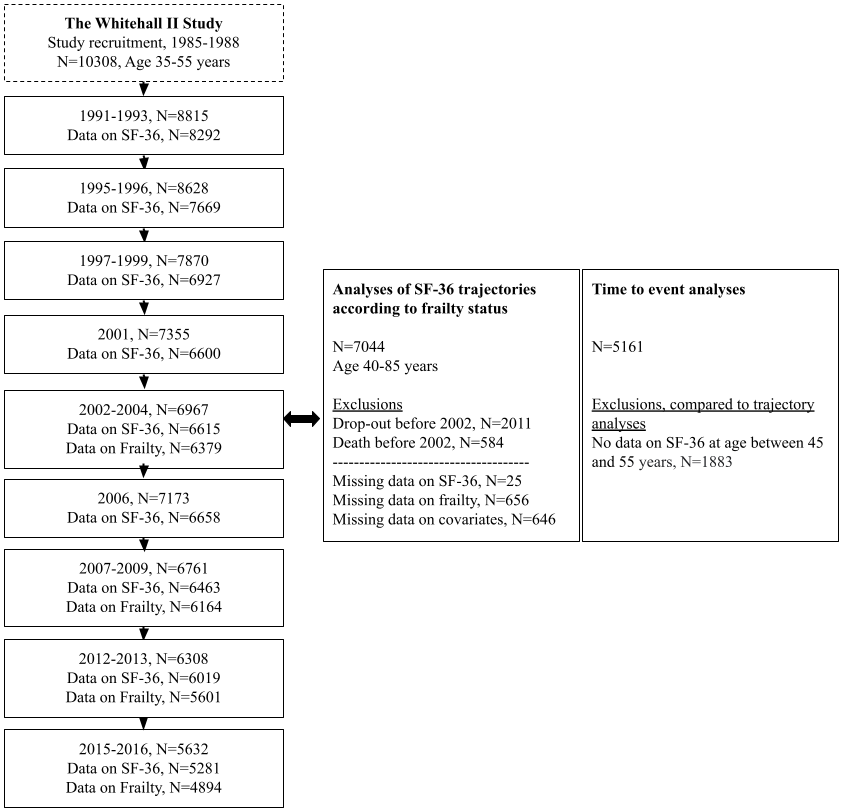
**

**Fig. S2. Trajectories of SF-36 component summary scores from age 40 to 85 years as a function of frailty status defined using Fried’s Frailty Phenotype (FFP). ***

**
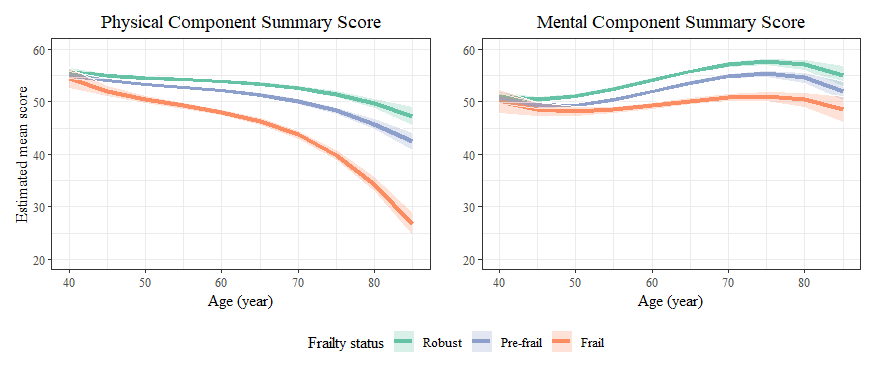
**

* Higher SF-36 scores reflect better health. Estimates from linear mixed models; analyses included age terms (age, age², and age^3^), sex, ethnicity, **time-varying covariates (**marital status, **occupational** position, alcohol consumption, smoking status, physical activity, fruit/vegetable consumption, body mass index, multimorbidity **status), FFP** status, and interaction of age terms with socio demographic measures and with **FFP** status.

**Fig. S3. Trajectories of SF-36 subscales from age 40 to 85 years as a function of frailty status defined using Fried’s Frailty Phenotype (FFP). ***

**
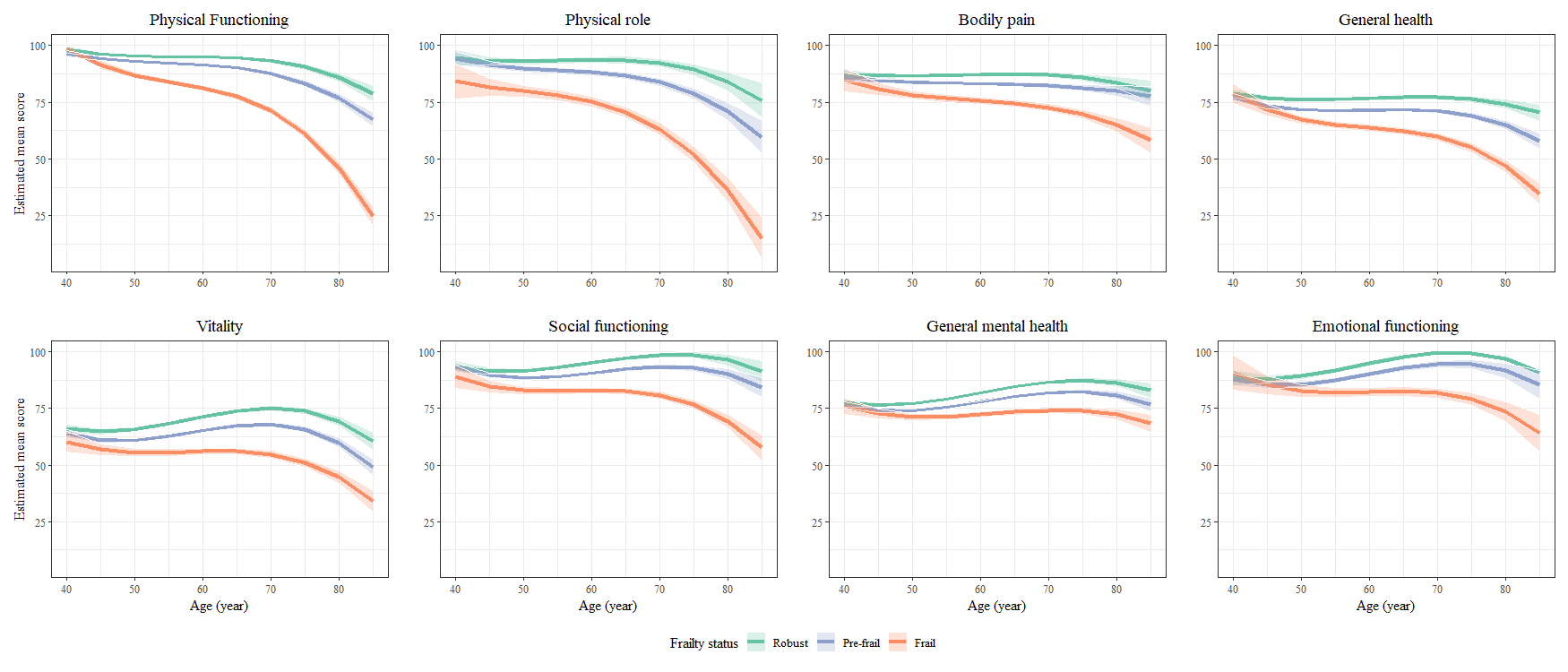
**

* Higher SF-36 scores reflect better health. Estimates from linear mixed models; analyses included age terms (age, age², and age^3^), sex, ethnicity, **time-varying covariates (**marital status, **occupational** position, alcohol consumption, smoking status, physical activity, fruit/vegetable consumption, body mass index, multimorbidity **status), FFP** status, and interaction of age terms with socio demographic measures and with **FFP** status.
